# Built-in Electric Field Induced Mechanical Property Change at the Lanthanum Nickelate/Nb-doped Strontium Titanate Interfaces

**DOI:** 10.1038/srep19017

**Published:** 2016-01-08

**Authors:** TeYu Chien, Jian Liu, Andrew J. Yost, Jak Chakhalian, John W. Freeland, Nathan P. Guisinger

**Affiliations:** 1 Department of Physics and Astronomy, University of Wyoming, Laramie, WY 82071, USA; 2 Department of Physics, University of Arkansas, Fayetteville, AR 72701, USA; 3 Department of Physics and Astronomy, University of Tennessee, Knoxville, TN 37996, USA; 4 Advanced Photon Source, Argonne National Laboratory, Argonne, IL 60439, USA; 5 Center for Nanoscale Materials, Argonne National Laboratory, Argonne, IL 60439, USA

## Abstract

The interactions between electric field and the mechanical properties of materials are important for the applications of microelectromechanical and nanoelectromechanical systems, but relatively unexplored for nanoscale materials. Here, we observe an apparent correlation between the change of the fractured topography of Nb-doped SrTiO_3_ (Nb:STO) within the presence of a built-in electric field resulting from the Schottky contact at the interface of a metallic LaNiO_3_ thin film utilizing cross-sectional scanning tunneling microscopy and spectroscopy. The change of the inter-atomic bond length mechanism is argued to be the most plausible origin. This picture is supported by the strong-electric-field-dependent permittivity in STO and the existence of the dielectric dead layer at the interfaces of STO with metallic films. These results provided direct evidence and a possible mechanism for the interplay between the electric field and the mechanical properties on the nanoscale for perovskite materials.

The mechanical properties of materials have been studied for decades, especially for simple metals and alloys^1^,^2^. In simple metals, the bonding is delocalized, the mechanical properties are determined by extrinsic factors such as impurities^3^, or grain boundaries (or grain sizes)^1^,^2^,^4^; while in covalent materials, the bonding is localized, the hardness is an intrinsic property^5^, which can be understood in terms of the electronic valence band structure^6^,^7^. It was also shown that the shorter bond length results in higher hardness^8^, while the higher bond density could enhance the fracture toughness^9^. Furthermore, for polar covalent crystals, besides the bond density and the bond length, a greater degree of covalent bonding is also an important factor to determine the high hardness^5^. In contrast to the hardness, which is related to the bond deformation, fracture toughness is related to the bond breaking in the crystal^10^. If the bond strength can be controlled at atomic scale by changing the bond length or density, the fracturing results are expected to change. Indeed, it has been shown and discussed for layered perovskite oxides that the bond length determines the fracture toughness intrinsically^11^.

Until recently, the electric field effects on the mechanical properties of materials at the nanoscale are relatively unexplored^12^,^13^,^14^,^15^,^16^. For nanoelectromechanical systems (NEMS), understanding the interplay between the electric field, the electron/charge transfer, and the mechanical behavior of the nanomaterial is especially crucial for NEMS applications, such as mass spectroscopy applications^17^,^18^,^19^,^20^. Fundamentally, we want to explore how charge transfer between two dissimilar materials alters the mechanical properties of the system and at what length scale? Here, we choose a non-polar perovskite oxide, Nb-doped SrTiO_3_ (Nb:STO), in contact with a metallic perovskite, LaNiO_3_^21^, to create a strong local electric field in the Schottky region near the interfaces. By utilizing cross-sectional scanning tunneling microscopy and spectroscopy (XSTM/S), we observe an altering of the fracture morphology that extends several nanometers into the Nb:STO substrate that extends the thickness of the interface with the LaNiO_3_ thin film. We believe that the structural change correlates with the formation of a built-in potential (Schottky barrier) that modifies the strength of Nb:STO within the depletion region, which is closely related to reports of a dielectric dead layer at the interfaces of Nb:STO with the metallic materials^22^.

## Sample Preparation

Crystalline LaNiO_3_ films were grown epitaxially by pulsed laser deposition on 1.0 at% Nb doped STO^23^. TiO_2_-terminated (001) STO single crystal substrates were prepared by the chemical wet-etch procedure. A KrF excimer laser (λ = 248 nm) was used to ablate a stoichiometry LaNiO_3_ target under 100 mTorr oxygen partial pressure. Growth temperature was 700 °C. After deposition, the samples were annealed at 600 °C under 1 atm of oxygen for one hour.

The LaNiO_3_/Nb:STO samples (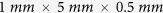) were then fractured at room temperature *in-situ* in ultra-high vacuum (UHV, base pressure was 3.0 × 10^−10^ mbar) prior to the XSTM/S measurements. The cross-sectional view of the interface was achieved by the controlled fracturing procedure reported earlier and is schematically illustrated in Fig. S1^24^. The set-point conditions for the STM measurements were a sample bias of 3.0 V and tunneling current of 20 pA, unless specified.

## Results and Discussions

**Figure 1:**
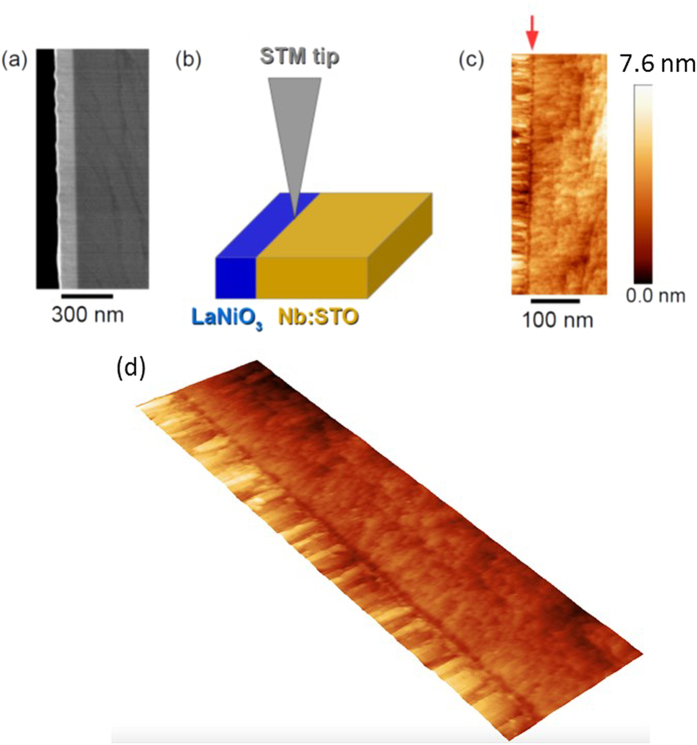
(**a**) SEM image; (**b**) The schematics of the XSTM measurement; and (**c**) XSTM topography, of LaNiO_3_/Nb:STO interfaces. The red arrow in (**c**) indicates the trench at the interfaces. (**d**) 3D plot of the topography.

The thickness of the LaNiO_3_ thin film was verified by a cross-sectional scanning electron microscope (XSEM) to be ~100 nm, as shown in Fig. 1(a). Figure 1(b) shows the schematics of the XSTM/S geometry, while Fig. 1(c) shows detailed topography of the fractured LaNiO_3_/Nb:STO interfaces measured at 53 K. The STM imaging reveals that the fractured Nb:STO substrate and the LaNiO_3_ film exhibits different topography, which clearly identifies the interface of interest as pointed out by the red arrow in Fig. 1(c). Upon closer inspection of the STM image, there appears to be a “trench-like” structure that uniformly spans the length of the LaNiO_3_/Nb:STO interfaces. This was verified by taking numerous measurements along the interfaces, confirming that the trench appears as a universal phenomenon following the fracture of the LaNiO_3_/Nb:STO system, as shown in 3D view of the topography in Fig. 1(d).

In order to understand the origin of the trench, we considered the following hypotheses or possible explanations: (1) lattice mismatch induced strain fracturing morphology; (2) intrinsic fracturing morphology of the materials; (3) imaging artifact due to LDOS at the interface; or (4) different fracture toughness compared with adjacent materials. A lattice mismatch induced strain fracturing morphology seems unlikely since trench-like features are not observed at La_2/3_Ca_1/3_MnO_3_/Nb:STO (LCMO/Nb:STO) interfaces^25^,^28^, which have similar lattice mismatch (*a* = 0.384 nm for LCMO)^29^ as LaNiO_3_/Nb:STO (*a* = 0.3837 nm for LaNiO_3_) and similar thickness of the thin film (150 nm in LCMO/Nb:STO and 100 nm here for LNO/Nb:STO)^30^.

**Figure 2:**
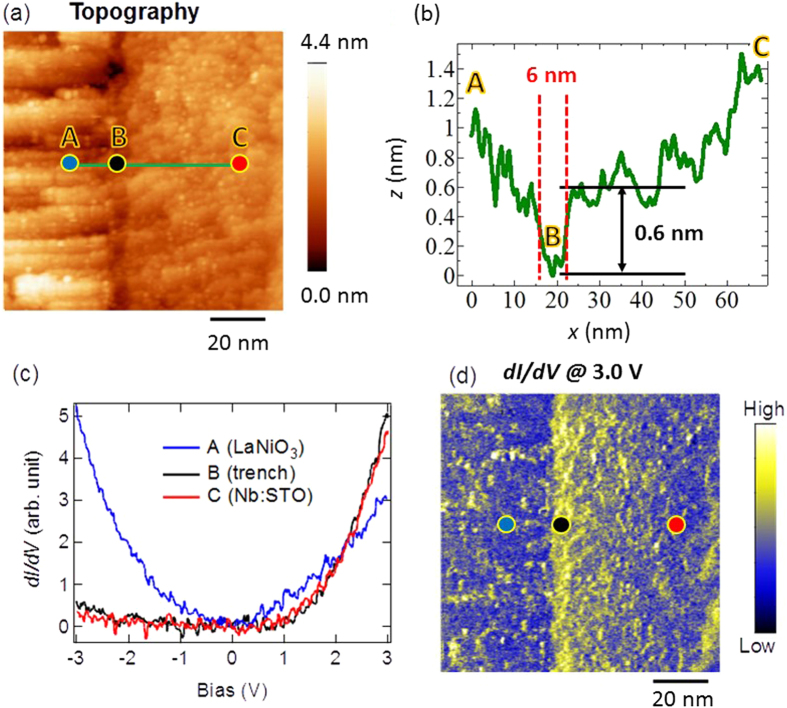
(**a**) Zoom-in XSTM topography. (**b**) Line profile along the green line in (**a**). (**c**) *dI/dV* spectra measured at locations A (LaNiO_3_), B (trench), and C (Nb:STO) indicated in (**a,b,d**). (**d**) *dI/dV* mapping measured simultaneously with the topography with bias of 3.0 V.

In order to fully understand the trench, we need to understand where the trench is located relative to the metallurgical interface. Does the trench lie within the Nb:STO region, the LNO region, or a combination of both. We further analyzed the trench by zooming in with XSTM/S to perform detailed tunneling spectroscopy near the interfaces. By analyzing the line profile (Fig. 2(b)) across the trench, as indicated by the green line in STM image of Fig. 2(a), the trench is determined to be ~6.0 nm wide and ~0.6 nm deep. Tunneling spectra were measured at points A (LaNiO_3_), B (trench), and C (Nb:STO), as indicated in Fig. 2(a,b,d), are plotted in Fig. 2(c). Spectrum A (LaNiO_3_) exhibits a spectrum without an energy gap near zero bias, indicating a metallic phase, which is consistent with the metallic phase of the LaNiO_3_ materials^21^. On the other hand, spectrum B (trench) is very similar to spectrum C (Nb:STO), with just a slight deviation, indicating that the material in the trench is Nb:STO. The fracturing behavior of Nb:STO has been reported^24^,^25^,^28^,^31^,^32^,^33^ without the observation of a trench, which indicates that its origin is unlikely due to an intrinsic fracturing morphology of the material.

The spectra B and C display an energy gap with the conduction band closer to the Fermi energy (zero bias), indicating an *n*-type semiconductor nature. At a sample bias of +3.0 V, a two-dimensional conductance map that spatially shows the local density of states (LDOS) and is shown in Fig. 2(d), which was measured concurrently with the topography in Fig. 2(a). At this energy we observe a higher LDOS in the trench that gradually decays into the substrate. This gradual change of the contrast is consistent with the slight deviation between spectrum B and C at 3.0 V. As will be discussed below, we believe that the increased LDOS in the trench at the interface results from the formation of the Schottky barrier at the LaNiO_3_/Nb:STO interface. Since the topographic trench has a clear boundary at ~6 nm away from the interfaces, the electronic DOS induced artifact scenario could be tested by examining if there exists an abrupt change in tunneling spectra around 6 nm away from the interfaces in Nb:STO.

**Figure 3:**
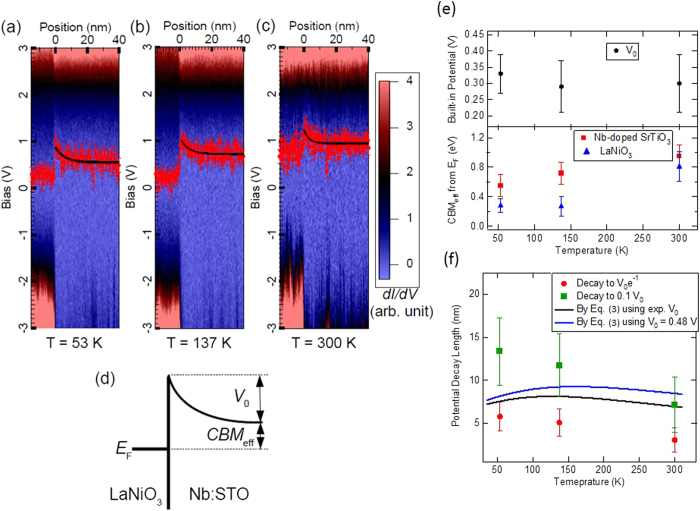
Point-by-point *dI/dV* measurements across the LaNiO_3_/Nb:STO interfaces (*0078* < 0: LaNiO3; x > 0: Nb:STO) at temperatures of (**a**) 53 K; (**b**) 137 K; and (**c**) 300 K. Red dots are the positions of CBMeff. Black curves are the fitting using Eq. 1. (d) Schematics of the Schottky region. (**e**) Built-in potential (*V*_0_) and the *CBM*_eff_ as functions of temperature. (**f**) Comparison between experimental potential decay length and the theoretical estimated depletion width (Eq. 3).

To visualize the Schottky barrier at the interface, the STS measurements were conducted point-by-point across the interfaces with pixel resolution of 0.5 nm. The spectrum averaged over 20 lines of point-by-point STS measurements across the interfaces at *T* = 53 K is shown in Fig. 3(a). The transition of the spectra across the LaNiO_3_/Nb:STO interface is very sharp, within 0.5 nm (note that one unit cell is ~0.39 nm). In contrast to previously proposed models on many oxide-based Schottky junctions^34^,^35^,^36^, no interfacial insulating barrier is visible. Effective conduction band minimum (*CBM*_eff_) of each spectrum at each position was extracted by the procedure described in the SI. Note that the *CBM*_eff_ is the bias where the signal falls below the measurable limit, rather than the real position of the *CBM*. However, the change of the *CBM*_eff_ is directly related to the change of the true *CBM* and/or the change of the conductance near Fermi energy [see discussion in SI]. The resulting *CBM*_eff_ at three different temperatures (53 K; 137 K; and 300 K) are plotted as red solid dots in Fig. 3(a–c). The Schottky band bending is clearly observed in Nb:STO side of the interfaces and is found to be temperature dependent. On the other hand, no band bending is shown in the *CBM*_eff_ in LaNiO_3_, which is expected for a metallic material. As a comparison, the band bending in Nb:STO is not observed in our previous studies of LCMO/Nb:STO interfaces^25^,^28^. We can rule out a LDOS induced imaging artifact for two reasons: (1) there is no abrupt electronic contrast found at the location of the edge of the topographic trench (~6 nm away from the interfaces); (2) the width of the trench measured at different temperatures does not change while the electronic contrast does (see Fig. 3(f)).

Lastly, let’s discuss the change of the fracture toughness as the possible mechanism. The appearance of the trench is a result of the fracturing process, it is likely that the formation of the trench is related to the different fracture toughness compared to the adjacent materials. The value of the change in the fracture toughness requires further investigation and proper experimental design, which is not in the scope of this study. However, with the present data we could argue that the trench is caused by lowering the fracture toughness with the following discussion. First, it has been shown that, using the same fracturing geometry and procedure for YBCO/LCMO superlattice system, LCMO is shown as trench (valley) compared to YBCO in contacted with it^28^. Though there is no report regarding the fracture toughness of LCMO and YBCO, Young’s modulus of YBCO (*E* = 141–370 GPa^37^) was reported to be higher than that of LCMO (*E* = ~120 GPa^38^). In general, fracture toughness increases as increasing the Young’s modulus^39^. It is likely that the smaller Young’s modulus in LCMO compared to that of YBCO indicates that the fracture toughness of LCMO is also smaller compared to that of YBCO. Following the same logic, the trench found in Nb:STO at the LaNiO_3_/Nb:STO interfaces indicates that the Nb:STO near the interfaces has lower fracture toughness compared to the Nb:STO substrate. The question is, what cause the change of the fracture toughness in the Nb:STO near the interfaces?

First of all, it is important to clarify that if there is inter-diffusion or oxygen vacancies near the interfaces? The LaNiO_3_/Nb:STO used in this study was synthesized by the same procedure and identical growth conditions as that of previous studies^40^,^41^. On the one hand, the TEM measurement indicated a coherent, defect-free film structure and an atomically sharp interface with no sign of cation interdiffusion near the LaNiO_3_/STO interfaces^40^; on the other hand, the resonant x-ray absorption measurements at Ni L-edge showed that the Ni^3+^ valence is well preserved, indicates no sign of inter-diffusion nor oxygen vacancy near the interface, since the high instability of the Ni^3+^ valence is extremely sensitive to oxygen deficiency as well as inter-diffusion^41^. In addition, although we have not fully characterized the effect of La diffusion, others have reported using cross-sectional transmission electron microscopy diffusion depths on the order of 2 nm or less for samples processed almost 100 **°**C hotter than our cases^42^. This diffusion length is much shorter than the observed trench and decay of the increased LDOS at the interface. Another point of view is the competing nature of the structural and electronic reconstructions at the interfaces. As discussed by Nakagawa *et al*.^43^, the imbalanced charge at dissimilar perovskite materials will likely to be compensated by either electronic or structural reconstructions. Which type of reconstruction will preferentially occur depends on the required energy. In general, the electronic reconstruction is easier (required lower energy) since electron is much mobile than ions. In the case of LaNiO_3_/Nb:STO, LaNiO_3_ is metallic while Nb:STO is slightly conductive in semiconducting phase. The electronic reconstruction should be much easier to be achieved compared to the structural reconstructions. In fact, this electronic reconstruction in our case is the formation of the Schottky region at the interfaces, which require charge redistributions.

In fact, the Schottky barrier profile (width and built-in potential) is the main difference between the LCMO/Nb:STO^25^ and LaNiO_3_/Nb:STO cases (see SI for detail comparison). There was no trench nor band bending observed in LCMO/Nb:STO case^25^; while here for LaNiO_3_/Nb:STO, the Schottky barrier is clearly observed. How does the built-in electric field change the fracturing toughness? Based on the different nature of the materials, different mechanisms were proposed to explain the electric field induced mechanical property change. For conductive materials, (1) change of the valence electron density^12^, and (2) the escape of vacancies from the grain interior to the grain boundary^44^,^45^, were proposed, however, without overall consensus^12^,^44^,^45^,^46^,^47^,^48^,^49^. For polar materials, such as ZnS, (3) the interactions between the electric field and the charged dislocations are responsible for the decreases of the hardness^15^. On the other hand, for non-polar materials, such as SiO_2_, (4) the change of the inter-atomic bond lengths due to the dielectric response (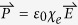) results in increasing the hardness^13^. As for piezoelectric materials, such as lead zirconate titanate (PZT), (5) the domain switching due to the electric field results in decreasing the fracture toughness^14^. First of all, Nb:STO is not a piezoelectric material nor a polar material, thus, mechanisms (3) and (5) could be ruled out. Mechanism (1) and (2) could also be ruled out since the change of the valence electron density and vacancies related defect states were not observed from the *dI/dV* spectra (only shifting was observed) near the interfaces. Similar to the non-polar dielectric material, the most possible mechanism here for Nb:STO is (4): the altered inter-atomic bond lengths in the Schottky region. The extraction of the detail profile of the Schottky barrier in LaNiO_3_/Nb:STO would be informative for further discussion.

To quantify the Schottky barrier, the *CBM*_eff_ in the Nb:STO side as function of distance away from the interfaces at three different temperatures, as shown in Fig. 3(a–c), were fit with an exponential decay function:


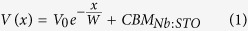


where *V*_0_ is the built-in potential, and *CBM*_Nb:STO_ is the *CBM*_eff_ of the Nb:STO far from the interfaces, both in the unit of V; *x* is the distance away from the interface, and *W* is the potential decay length, both in the unit of nm. *V*_0_ and *CBM*_Nb:STO_ are also defined in the schematics of the Schottky barrier in Fig. 3(d). The results of the fitting parameters, *W, V*_0_ and *CBM*_Nb:STO_ in Eq. 1, as well as 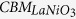, are plotted as a function of temperature in Fig. 3(e,f). Since the band structures are not expected to be changed dramatically upon the change of the temperature, the up shifts of the *CBM*_Nb:STO_ and the 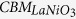 as the temperature increases from 53 K to room temperature are most likely related to the reduction of the conductance near Fermi energy, which is closely related to the higher resistivity observed at room temperature compared to low temperature in both LaNiO_3_ and similar doping level of Nb:STO^21^,^50^,^51^. Contrary to the shifting of the *CBM*_eff_ in LaNiO_3_ and Nb:STO, the spatial evolution of the *CBM*_eff_ in Nb:STO near interfaces is the band bending caused by the built-in electric field in the Schottky barrier.

For Schottky barrier, two major quantities are important to describe the potential profile across the interfaces: built-in potential, *V*_0_, and depletion width, *W*. As shown in Fig. 3(e), the built-in potential, *V*_0_, is temperature independent: 0.3 ± 0.1 V. In semiconductor physics, the built-in potential for metal/*n*-type semiconductor interfaces could be estimated by:


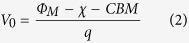


where 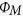 is the metal work function, which is 4.6 eV for LaNiO_3_^52^; 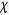 is the electron affinity of the semiconductor, which was reported to vary in the range of 3.9–4.3 eV for STO^53^,^54^,^55^,^56^; *CBM* is the conduction band minimum relative to the Fermi energy, which was reported to be 0.02 eV for Nb 1.0 at% doped STO^36^; and *q* is the electron charge. With the information, the built-in potential, *V*_0_, at the LaNiO_3_/Nb:STO interface is estimated to be in the range of 0.28–0.68 V (or 0.48 ± 0.20 V), which is consistent with the measured value (*V*_0_ = 0.3 ± 0.1 V) within the error bar.

Now, let us move our attention to the depletion width. The results of the potential decay length, *W*, as defined in Eq. 1, as a function of temperature is plotted as red circular dots in Fig. 3(f). The potential decay length, *W*, increases from ~3 nm at 300 K to ~6 nm at 53 K. However, the depletion width is traditionally defined as the width that the *CBM* decreases to the same value in the semiconductor far from the interfaces. Since the exponential function used in Eq. 1 will only decay to zero when the distance is infinity, instead, we investigated the length when the potential difference (*V*(*x*) – *CBM*_Nb:STO_) decays to 0.1 *V*_0_ and define this as the “effective depletion width” plotted as solid green squares in Fig. 3(f). The resulting effective depletion width increases from ~7 nm at 300 K to ~13.5 nm at 53 K. Unlike regular semiconductors, to estimate the temperature-dependent depletion width in Nb:STO, the strong temperature and electric field dependence of the permittivity, which could be described by 
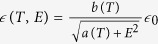
 in STO^36^, has to be taken into account:


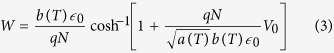


where *N* is the carrier concentration, which is 1.6 × 10^20^ cm^−3^ for 1 at% Nb doped STO;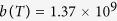 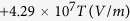; 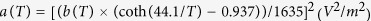
36 Besides these information, *V*_0_ is needed to estimate the expected depletion width in Nb:STO. To have a full comparison, both the experimental mean *V*_0_ (0.3 V) and the theoretical mean value (0.48 V, as deduced above) are used and the resulting *W* are plotted in Fig. 3(f). The predicted depletion widths agree well with the experimental effective depletion widths at room temperature. However, in contrast to the monotonically increase of the experimental effective depletion width with decreasing temperature, the predicted depletion widths show a non-linear behavior within the temperature range studied. The discrepancy might origin from that *a(T)* and *b(T)* were extracted from bulk experiments; while the observations here were in nanometer-scale, which might deviate from the bulk values. This postulation deserves further investigation. Interestingly, the effective depletion width (~7 nm at room temperature, which is the fracturing temperature) is comparable with the width of the topological trench (~6 nm) in Fig. 2(a), which supports the picture that the electric field in Schottky region induced fracturing results. Note that the minimum unit for breaking the Nb:STO crystal is one atomic thickness (which is half unit cell for Nb:STO). The trench depth of 0.6 nm is 1.5 unit cell thick, which is three times of the smallest possible breaking unit.

**Figure 4:**
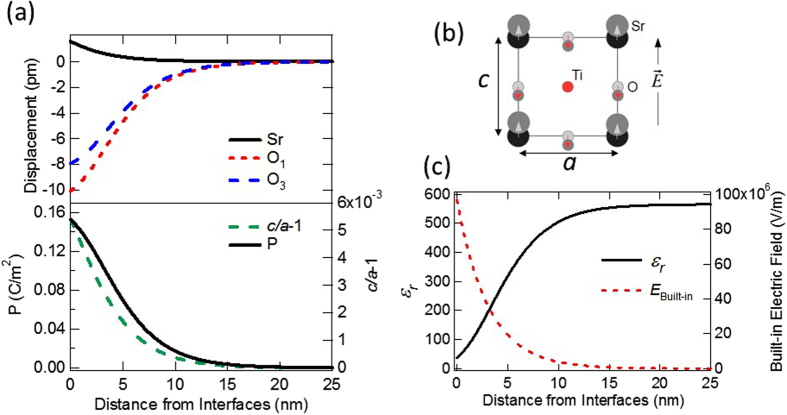
(**a**) Calculated relative atomic displacements of Sr, O_1_, and O_3_ (top panel); *c/a*-1 and polarization density strength (bottom panel) at room temperature as functions of the distance from the interfaces. (**b**) Schematics of the relative atomic displacement with respect to Ti atom upon the application of the electric field. (**c**) Extracted electric field from experimental data and calculated dielectric constant as function of distance from the interfaces.

The electric field induced inter-atomic bond length change is directly related to the polarization density (or equivalently to the permittivity/dielectric constant) of the dielectric materials. As illustrated in Fig. 4(b), upon the application of the electric field, atoms with different valences displace slightly from their original position, thus changing the inter-atomic bond length. The polarization density, 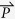, could be calculated by 
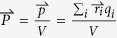
, where 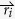 and 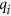 are the position vector and charge of atom *i* in an unit cell, *V* is the volume of an unit cell. Note that the polarizability of each individual atom is ignored here for simplicity. For linear dielectric materials, 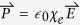; where 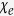 is the electric susceptibility, which is related to the permittivity as: 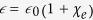. In other words, if the strong electric field in the Schottky barrier altered the inter-atomic bond length, the dielectric constant near the interfaces would be changed as well. In fact, the dielectric dead layer at the interfaces of STO and metallic films^57^,^58^,^59^, as well as the thickness dependent dielectric constant^57^,^59^ were reported.

Based on the experimental Schottky barrier profile, the electric field, the change of the bond length and the dielectric constant as function of the distance away from the interfaces could be determined. First, the electric field as function of distance in the Schottky region is extracted from the experimental *V(x)* shown in Fig. 3(c) by 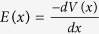 at room temperature, as shown in Fig. 4 (c). The relative atomic displacements (Sr, O_1_(in-plane oxygen), and O_3_(apical oxygen) relative to Ti) and *c/a*-1 (*c* and *a* are lattice constants along [001] and [100] directions, respectively) in Nb:STO near the interfaces are then determined and shown in Fig. 4(a) with the information of the electric-field dependent values^60^. The unit cell volume, *V*, changes upon the application of the electric field was considered by assuming lattice constant *a* is not changed while *c* changes following the reported *c/a*-1 curve by Naumov *et al*.^60^. Note that the direction of the displacements are defined in Fig. 4(b), where “up” is defined as “positive” in Fig. 4(a). Sr has same sign of valence as Ti, the displacement of Sr is small (around one order of magnitude smaller) compare to that of oxygen, which has opposite sign in valence. From Fig. 4(a), the Sr-O bond length change could be estimated to be in the order of 10 pm in the Schottky region. Compared to the lattice constant of STO (0.39 nm), this bond length change is ~2.5 %, which is comparable to the high strain scenario for epitaxial thin film growth.

With the atom displacement information, the polarization density as function of the distance is calculated by 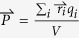 and shown in Fig. 4(a). To determine the dielectric constant, one has to considered that STO is a non-linear dielectric material with electric field dependent permittivity^36^. Instead of the linear response equation, the dielectric constant should be extracted from the electric-field-dependent polarization density strength, 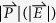, by


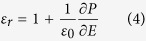


The electric-field-dependent polarization density strength, 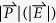, could be calculated from the electric-field-dependent relative atomic displacements information reported by Naumov *et al*.^60^ through 
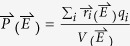
. The resulting dielectric constant as function of the distance is shown in Fig. 4 (c), where the dielectric dead layer near the interfaces is revealed. This finding is consistent with the mechanism reported by Stengel *et al*. that the origin of the dielectric dead layer is an intrinsic properties of STO due to the electrostatic screening effect^22^.

## Summary

In summary, by XSTM/S technique, the built-in potential in the Schottky region and topographic trench due to the fracturing process are observed with similar length scale: ~6–7 nm. The correlation between the topographic trenches and the electric field is established, indicating the importance of the electric field on the mechanical properties in nanoscale. Furthermore, possible mechanisms are discussed and the electric field induced inter-atomic bond length change is argued as the most plausible mechanism. The same picture points toward the connections with the reported dielectric dead layer at the interfaces of STO and metallic films. The observations here show that the Schottky region in complex oxides might play an important role on determining mechanical and electronic properties in nanoscale.

## Additional Information

**How to cite this article**: Chien, T.Y. *et al*. Built-in Electric Field Induced Mechanical Property Change at the Lanthanum Nickelate/Nb-doped Strontium Titanate Interfaces. *Sci. Rep*. **6**, 19017; doi: 10.1038/srep19017 (2016).

## Supplementary Material

Supplementary Information
