# Supplementary material for: Built-in Electric Field Induced Mechanical Property Change at the Lanthanum Nickelate/Nb-doped Strontium Titanate Interfaces
Source: Sci Rep. 2016 Jan 8;6:19017. doi: 10.1038/srep19017 (PMC4705578; doi:10.1038/srep19017)
Supplement: Supplementary Information [file srep19017-s1.pdf]

# Built-in Electric Field Induced Mechanical Property Change at the Lanthanum Nickelate/Nb-doped Strontium Titanate Interfaces
# Supplementary Information

TeYu Chien[1]*, Jian Liu[2,3], Andrew J. Yost[1], Jacques Chakhalian[2], John W. Freeland[4], and Nathan P. Guisinger[5]

[1]Department of Physics and Astronomy, University of Wyoming, Laramie, WY 82071, USA
[2]Department of Physics, University of Arkansas, Fayetteville, AR 72701, USA
[3]Department of Physics, University of California, Berkeley, CA 94720, USA
[4]Advanced Photon Source, Argonne National Laboratory, Argonne, IL 60439, USA
[5]Center for Nanoscale Materials, Argonne National Laboratory, Argonne, IL 60439, USA

* tchien@uwyo.edu

## Fracturing Method and Procedure

For cross-sectional scanning tunneling microscopy and spectroscopy (XSTM/S) measurements, a reliable and reproducible fracturing method and procedure is needed for high quality interfaces imaging.[1-8] Figure S1(a) shows the schematics of the sample holder for XSTM/S measurements. The sample holder is modified from a regular STM sample plate by adding two metal (stainless steel) blocks as clamps. The clamps are fixed by screws on the sample plate and the samples are held by another set of screws to hold the other metal block to the one mounted on the sample plate. After the oxide thin film were deposited on Nb:STO substrate (usually with dimension of $10 \times 10 \times 1\, mm^3$), sample was further cut into desired dimension of $8 \times 8 \times 1\, mm^3$ . The controllable fracture process is done with an initial notch served as the weak point for controlling the location of the cracking front. The notch is created by cutting the sample half way through the sample. The key for creating high quality interfaces for XSTM/S measurements is to fracture from the side of the film, as shown in Fig. S1(b). The samples are then mounted sideway for side fracturing, and the fracturing process is done by holding the bottom half of the sample by the clamps while having the top portion is moving against a sturdy metal cleaver. Although we expect to see hump on the top portion of the sample if a trench is observed in the bottom portion (the one that is measured by XSTM/S), after the fracture, the top portion of the sample dropped into the UHV chamber and cannot be recovered for further study.

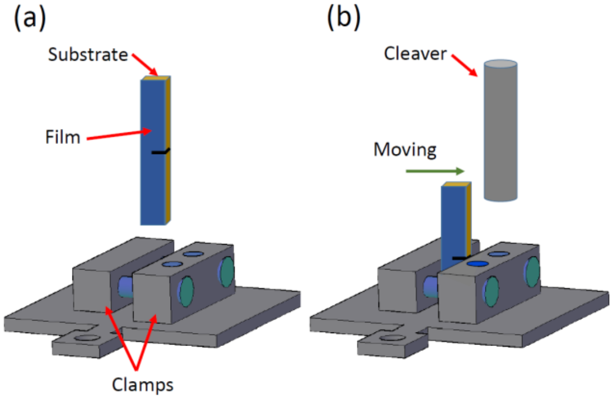


*Figure S1. Sample holder for Cross-sectional Scanning tunneling microscopy measurements. (a) the way of sample mounting; (b) side fracturing process.*

## Comparison with LCMO/Nb:STO and $(YBCO/LCMO)_n$/Nb:STO

The fractured interfaces of $LaNiO_3$/Nb:STO (LNO/Nb:STO) are very different from that of $La_{2/3}Ca_{1/3}MnO_3$/Nb:STO (LCMO/Nb:STO),[6] in both topography and electronic band bending. The topography in LCMO/Nb:STO exhibits no trench;[6] while that in LNO/Nb:STO, a trench is clearly shown with ~6 nm width and ~0.6 nm depth (see Fig. 2 in main text). On the other hand, the band bending in LNO/Nb:STO was revealed by measuring *dI/dV* spectra point-by-point across the interfaces (see Fig. 3 in main text); while the spatial evolution of the *dI/dV* spectra across the LCMO/Nb:STO interfaces does not show any sign of band bending.[6] Same conclusion could be drawn with the *dI/dV* mapping at tunneling bias of 3.0 V for both LCMO/Nb:STO and LNO/Nb:STO interfaces that a gradual contrast change is seen in

LNO/Nb:STO interfaces and no contrast change in LCMO/Nb:STO interfaces were observed.[6] Furthermore, the results for YBCO/LCMO superlattices on Nb:STO showed clear topography difference between YBCO and LCMO.[1] The topographic height difference is ~2 nm and is argued to be caused by the different fracture toughness in the main text.

## Definition and the Meaning of the Effective CBM

To quantitatively analyze the observed *dI/dV* spectra across the interfaces, we defined the effective CBM, $CBM_{eff}$, for further discussion. Here is how to determine the $CBM_{eff}$: (1) find the statistical average conductance, $(dI/dV)_{ave}$, and the standard deviation of the conductance, $(dI/dV)_{sdv}$, in the flat noise region (blue regions in Fig. 3(a)-(c)); (2) at each position, the $CBM_{eff}$ is defined to the onset bias where the *dI/dV* signal averaged over a vicinity of ±0.3 V region (which equivalent to averaging over 21 data points in our data set) is higher than $(dI/dV)_{ave}$ + $(dI/dV)_{sdv}$. To clearly explain this procedure, we created a simulated *dI/dV* spectra with white noise and used them to discuss the effects of the true *CBM* shifting and change of the conductance near Fermi energy. The simulated *dI/dV* spectrum in Fig. S2(a) is created with the following equation plus a white noise:

$$\frac{dI}{dV}(V) = \begin{cases} 0.35e^{|V|} & for\ V \geq \frac{E_g}{2} \\ 0 & for\ V < \frac{E_g}{2} \end{cases} \quad \text{(S-1)}$$

Where $E_g$ is set to be 2.10 eV. The white noise is simulated with a random number within (-0.5, 0.5) range. Following the procedure described above, the values of $(dI/dV)_{ave}$ and $(dI/dV)_{sdv}$ are shown in the figures with dashed lines. The $CBM_{eff}$ is determined to be at ~1.28 V, which is ~0.23 V higher than the $CBM_{true}$. The location of the $CBM_{true}$ cannot be determined due to the probing limitation of the *dI/dV* signal.

Now, let's discuss two different scenario: (1) shift of $CBM_{true}$; and (2) change of the conductance (*dI/dV*) without shifting of the

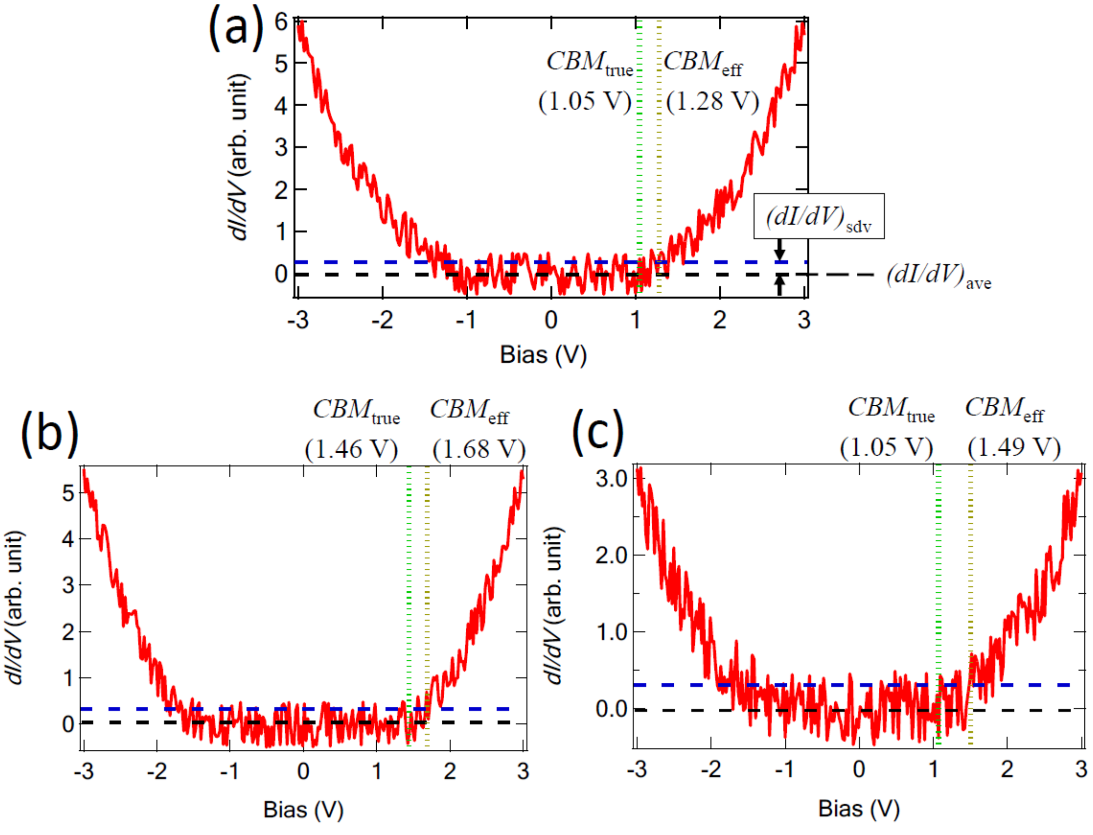


*Figure S3. Simulated dI/dV spectra for explaining the $CBM_{eff}$. (a) dI/dV spectrum generated from Eq. S-1 with $E_g$ = 2.10 eV; (b) dI/dV spectrum generated from Eq. S-1 with $E_g$ = 2.92 eV; and (c) dI/dV spectrum generated by multiply 0.5 to the one in (a) before adding the white noise.*

$CBM_{true}$. For first scenario, we created a simulated *dI/dV* spectrum using Eq. S-1 with just changing the $E_g$ to 2.92 eV. Following the same procedure, the $CBM_{eff}$ is found to be ~0.22 eV higher than the $CBM_{true}$. Note that the deviation between $CBM_{eff}$ and $CBM_{true}$ in this case is the same as that of the original one (Fig. S2(a)).

On the other hand, for the second scenario, the simulated *dI/dV* spectrum was created by multiply 0.5 to the Eq. S-1 followed by adding the white noise. In this case, $E_g$ remains the same as 2.10 eV. Following the same procedure, the $CBM_{eff}$ is determined to be 1.49 V, which is ~0.44 V higher than the $CBM_{true}$, which is not shifted compared to the case in Fig. S2(a).

In short, the $CBM_{eff}$ is different from the $CBM_{true}$ due to the signal limitation of the STS measurements. Two scenarios are discussed to have the effect of shifting the $CBM_{eff}$: (1) the location of the $CBM_{true}$ is shifting; (2) the conductance (*dI/dV*) near the measuring limitation is changing.

References:


1. TeYu Chien, L. F. Kourkoutis, J. Chakhalian, B. Gray, M. Kareev, N. P. Guisinger, D. A. Muller, and J. W. Freeland, "Visualizing short range charge transfer at the interfaces between ferromagnetic and superconducting oxides," *Nature Commun.* **4**, 2336 (2013).
2. TeYu Chien, J. Chakhalian, J. W. Freeland, and N. P. Guisinger, "Cross-sectional scanning tunneling microscopy applied to complex oxide interfaces", *Adv. Funct. Mater.* **23**, 2565 (2013).
3. TeYu Chien, J. W. Freeland, and N. P. Guisinger, "Morphology control of Fe films using ordered termination on $SrTiO_3$ surfaces", *Appl. Phys. Lett.* **100**, 031601 (2012).
4. TeYu Chien, N. P. Guisinger, and J. W. Freeland, "Cross-sectional scanning tunneling microscopy for complex oxide interfaces", *Proc. SPIE* **7940**, 79400T (2011).
5. TeYu Chien, N. P. Guisinger, and J. W. Freeland, "A survey of fractured $SrTiO_3$ surfaces: from the micrometer to nanometer scale", *J. Vac. Sci. & Technol. B* **28**, C5A11-C5A13 (2010).
6. TeYu Chien, J. Liu, J. Chakhalian, N. P. Guisinger, and J. W. Freeland, "Visualizing nanoscale electronic band alignment at the $La_{2/3}Ca_{1/3}MnO_3$/Nb:$SrTiO_3$ interface", *Phys. Rev. B* **82**, 041101(R) (2010).
7. N. P. Guisinger, T. S. Santos, J. R. Guest, TeYu Chien, A. Bhattacharya, J. W. Freeland, and M. Bode, "Nanometer-scale striped surface terminations on fractured $SrTiO_3$ surfaces", *ACS Nano* **3**, 4132-4136 (2009).
8. TeYu Chien, T. S. Santos, M. Bode, N. P. Guisinger, and J. W. Freeland, "Controllable local modification of fractured Nb-doped $SrTiO_3$ surfaces", *Appl. Phys. Lett.* **95**, 163107 (2009).
